# Recent progress in the development of steroid sulphatase inhibitors – examples of the novel and most promising compounds from the last decade

**DOI:** 10.1080/14756366.2020.1758692

**Published:** 2020-05-04

**Authors:** Mateusz Daśko, Sebastian Demkowicz, Karol Biernacki, Olga Ciupak, Witold Kozak, Maciej Masłyk, Janusz Rachon

**Affiliations:** a Department of Inorganic Chemistry, Faculty of Chemistry, Gdansk University of Technology, Gdansk, Poland; b Department of Organic Chemistry, Faculty of Chemistry, Gdansk University of Technology, Gdansk, Poland; c Department of Physical Chemistry, Faculty of Chemistry, University of Gdansk, Gdansk, Poland; d Department of Molecular Biology, Faculty of Biotechnology and Environment Sciences, The John Paul II Catholic University of Lublin, Lublin, Poland

**Keywords:** Steroid sulphatase, STS inhibitors, steroids, multitargeting agents

## Abstract

The purpose of this review article is to provide an overview of recent achievements in the synthesis of novel steroid sulphatase (STS) inhibitors. STS is a crucial enzyme in the biosynthesis of active hormones (including oestrogens and androgens) and, therefore, represents an extremely attractive molecular target for the development of hormone-dependent cancer therapies. The inhibition of STS may effectively reduce the availability of active hormones for cancer cells, causing a positive therapeutic effect. Herein, we report examples of novel STS inhibitors based on steroidal and nonsteroidal cores that contain various functional groups (e.g. sulphamate and phosphorus moieties) and halogen atoms, which may potentially be used in therapies for hormone-dependent cancers. The presented work also includes examples of multitargeting agents with STS inhibitory activities. Furthermore, the fundamental discoveries in the development of the most promising drug candidates exhibiting STS inhibitory activities are highlighted.

## 1. Introduction

Cancer is among the leading causes of death. According to the *International Agency for Research on Cancer* estimates in 2018, there were more than 18 million new cases and 9.5 million tumour-related deaths worldwide^1^. Additionally, the *National Cancer Institute* (NCI) expects that the number of new cancer cases will have risen to approximately 23.6 million per year by 2030. The NCI warns that this disease will be diagnosed in approximately 38.4% of men and women during their lifetimes. The most common types are breast, lung, and bronchus, prostate and colorectal tumours, and they account for almost 50% of all new cancer cases. Moreover, lung and bronchus, colorectal, pancreatic, and breast cancers are responsible for nearly 50% of all deaths. The estimates for 2019 indicate that almost 270,000 and 175,000 patients will be diagnosed with breast and prostate tumours, respectively, and more than 41,000 (breast) and 31,000 (prostate) deaths will occur from these diseases in the United States^2^. It is known that most cancers show a hormone-dependent nature in their early stages (e.g. more than 90% of breast cancer cases are initially hormone-dependent)^3^. Therefore, the *World Health Organisation* (WHO) describes biologically active hormones (androgens and oestrogens) as the main cancer growth stimulants. Considering the aforementioned facts, the application of drugs that can effectively reduce concentrations of active hormones should be the basis of modern therapies^4^.

**Scheme 1.**
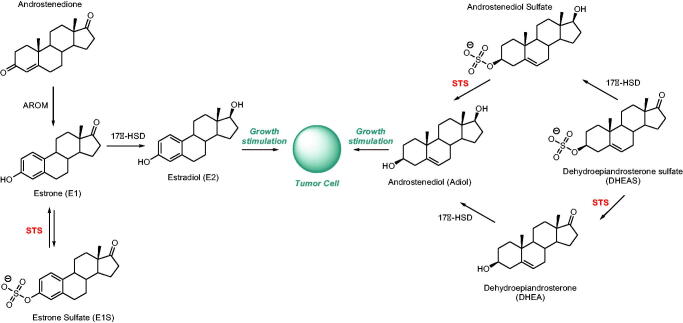
The biosynthesis pathway for steroids with oestrogenic properties.

**Figure 1.**
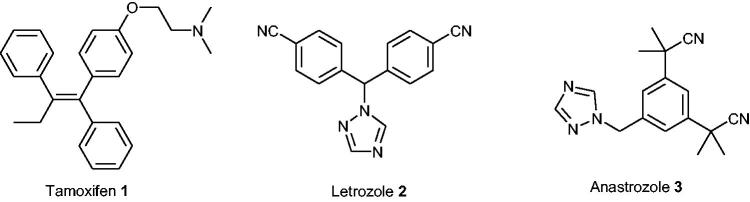
Chemical structures of *Tamoxifen* **1**, *Letrozole* **2,** and *Anastrozole* **3**.

The hormone signalling pathway is a well-established target for the development of hormone-dependent cancer drugs (e.g. breast cancer)^5^. For example, the clinically used drug *Tamoxifen* **1** (Figure 1) acts as a selective oestrogen receptor modulator (SERM). In contrast, chemotherapeutics, which may influence the hormone formation process, are also of high therapeutic importance. The biosynthesis of active steroids (e.g. oestradiol [E2] and androstenediol [Adiol]) in cancer tissues mainly depends on the following three enzymatic pathways: aromatase (AROM), 17β-hydroxysteroid dehydrogenase (17β-HSD), and steroid sulphatase (STS) (Scheme 1)^6^. For example, currently used *Letrozole* **2** and *Anastrozole* **3** (Figure 1) block the conversion of androgens to oestrogens *via* inhibition of the AROM complex. However, therapies using the described above drugs often turn out to be unsatisfactory and result in the development of resistance, leading to relapses in tumour progression^7–10^. In light of recent research indicating that sulphation/desuphfation process disorders may be responsible for numerous pathologies^11^, another enzyme implicated in the steroidogenesis process, STS, is becoming a new interesting molecular target in the development of novel and effective hormone-dependent cancer treatment methods. In contrast to aromatase, STS activity is present in most cancer cases (e.g. STS expression is detected in 90% of breast tumours)^12^. Furthermore, it has been noticed that STS mRNA levels in malignant tissues have been higher than in normal breast tissues in 87% of tested patients^13^.

STS belongs to a group of 15 human sulphatases^6^. This protein consists of 587 amino acid residues and is encoded by the *STS* gene. STS is found ubiquitously throughout the body, what is strictly related to its involvement in numerous physiological and pathological processes^14^. This enzyme is mainly localised in skin, fallopian tubes, testis, ovary, adrenal glands, brain, foetal lung, endometrium, aorta, kidneys, bones, placenta, and breasts^15^. STS catalyses the hydrolysis of steroid sulphates (including oestrone sulphate [E1S] and dehydroepiandrosterone sulphate [DHEAS]) to their unsulphated derivatives (oestrone [E1] and dehydroepiandrosterone [DHEA], respectively) (Scheme 1)^16^^,^^17^. E1 and DHEA may be subsequently transformed into bioactive oestrogens and androgens (e.g. E2 and Adiol, respectively), which are responsible for the stimulation of hormone-dependent cancer cell proliferation^18^. Considering the aforementioned facts, STS plays a pivotal role in breast cancer tumourigenesis and is, therefore, an extremely attractive molecular target for the development of hormone-dependent cancer therapies.

**Figure 2.**
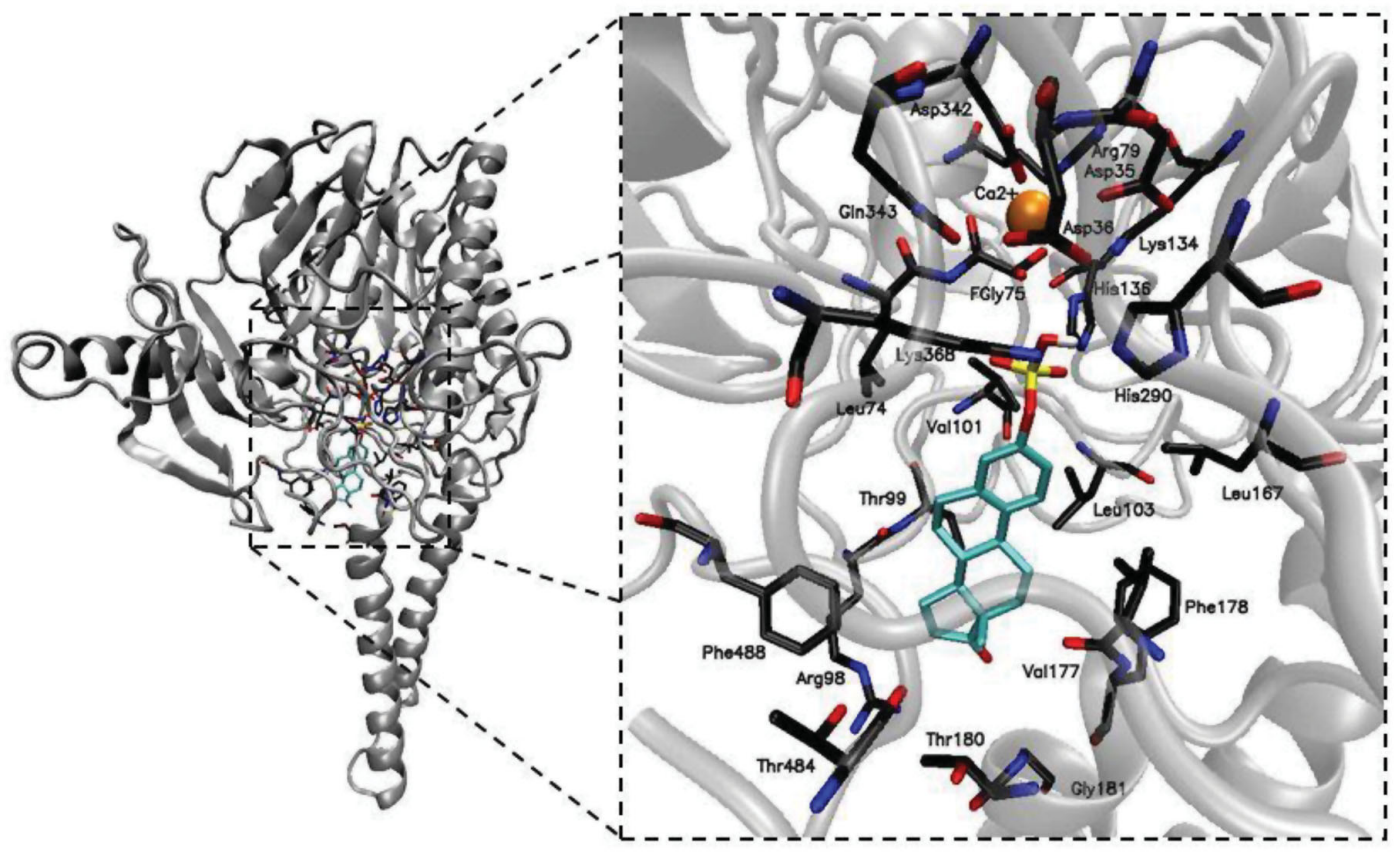
The structure of STS with its natural substrate (E1S) bound to the active site.

The crystallographic structure of STS is known^19^. It is composed of a globular domain with polar characteristics and a stem domain consisting of two antiparallel hydrophobic helices that resemble a mushroom structure. The active site is located in a cavity on the border of polar and hydrophobic domains of the enzyme^20^. STS demonstrates a high similarity to arylsulphatase A (ARSA) and B (ARSB). The topology of active sites of all three enzymes is very similar. One of the characteristic features of all sulphatases is a posttranslational modification within the active site involving the conversion of cysteine to a formylglycine residue (fGly)^21^. In the absence of substrate, the catalytic region of human STS consists of a sulphated fGly residue in its *gem*-diol form, which is coordinated to a calcium ion. Moreover, the enzyme’s active site is composed of nine other catalytically important amino acid residues, including Asp35, Asp36, Arg79, Lys134, His136, His290, Asp342, Gln343, and Lys368. However, when a natural substrate (e.g. E1S) associates with the STS active site (Figure 2), its steroidal core becomes surrounded by other amino acid residues located in the binding region (Leu74, Arg98, Thr99, Val101, Leu103, Leu167, Val177, Phe178, Thr180, Gly181, Thr484, and Phe488) and interacts with them *via* hydrophobic interactions^14^.

**Scheme 2.**
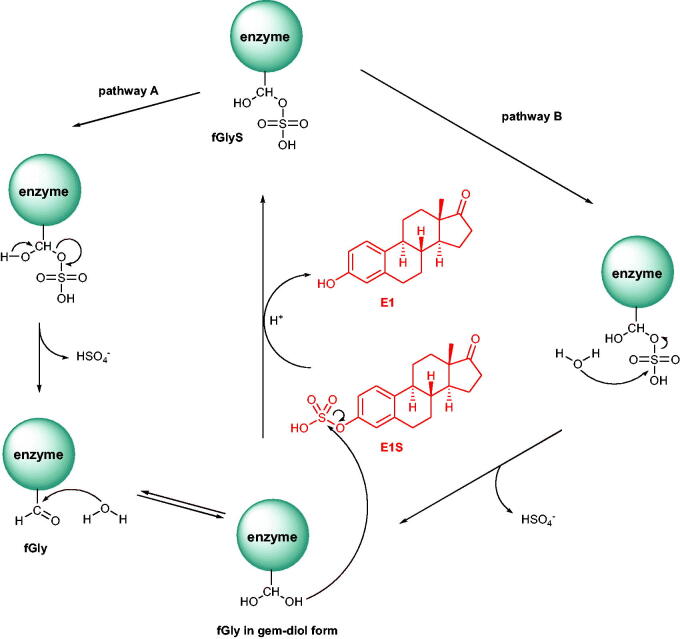
Two putative mechanisms of action for STS (pathways A and B).

Because the fGly residue, in its *gem*-diol form, is involved in the hydrolysis of sulphate substrates in ARSA and ARSB, a mechanism of enzymatic reaction catalysed by STS may proceed according to two putative pathways (Scheme 2). Pathway A starts with the decomposition of formylglycine sulphate (fGlyS) into a sulphate anion and fGly, which then reacts with a water molecule furnishing the hydrated formylglycine form. In contrast, pathway B assumes the straightforward nucleophilic attack of a water molecule on the fGlyS sulphur atom, giving rise to the fGly *gem*-diol form, which subsequently reacts with E1S to release E1 *via* S_N_2 attack of one of the hydroxyl groups^14^.

Studies focussed on the development of effective STS inhibitors lacking adverse side effects as drug candidates have been carried out for over 30 years. The first fruitful 1990–1999 decade was successfully continued during the years of 2000–2010. In that time, many scientific papers devoted to the design, synthesis, and biological evaluation of compounds based on steroidal or nonsteroidal cores were published. Those years’ achievements have been well summarised in a few scientific reviews^6^^,^^12^^,^^14^^,^^18^^,^^22–25^. The unique significance of these two decades highlights the fact that almost thorough clinical progress is strictly associated with a few STS inhibitors discovered at that time (e.g. *E2MATE*, *Irosustat* [also known as *667-COUMATE*, *STX64* and *BN-83495*])^26–29^.

The results of some clinical trials dedicated to known STS inhibitors seem to be promising and such compounds have currently a great chance to be used in the treatment of several hormone-dependent types of cancer (especially, hormone-dependent breast cancer [HDBC]). However, more clinical trials involving significantly greater number of patients are definitely required. Furthermore, it is worth noting that to date, none of the developed STS inhibitors has reached the pharmaceutical market and therefore, there is still place for the development of novel drug candidates (e.g. for compounds, which mechanism of action is based on some innovative multi-targeting approaches). The need for these studies results from the unique and extremely important role of STS in biosynthesis of active steroids mentioned above. Furthermore, according to the opinions presented in many scientific papers and evidences confirming the key role of STS enzyme in the process of growth and development of hormone-dependent cancers, the intensive search for new and effective STS inhibitors seems to be fully justified. Therefore, a summary of the last years’ achievements in the identification and synthesis of novel derivatives demonstrating STS inhibitory properties may be valuable for future researchers.

## 2. Steroidal STS inhibitors

**Figure 3.**
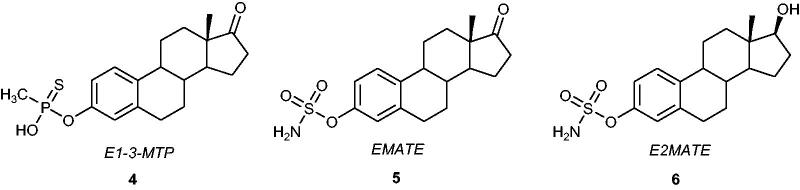
Chemical structures of *E1-3-MTP* **4**, *EMATE* **5,** and *E2MATE* **6**.

Initially, the development of STS inhibitors was based on the synthesis of steroid analogues. For example, replacement of the sulphate group in E1S with phosphonate and thiophosphonate moieties led to obtain one of the first potent STS inhibitors, oestrone 3-*O*-methylthiophosphonate **4** – *E1-3-MTP* (Figure 3)^30^. In MCF-7 breast cancer cells, *E1-3-MTP* competitively inhibited STS activity by 52 and >98% at 100 and 10 µM concentrations, respectively^31^. In the early 90 s, the research group of *Prof. Michael Reed* and *Dr. Atul Purohit* at Imperial College London, and *Prof. Barry Potter* at Bath University pioneered the STS inhibitors field including a new series of STS inhibitors based on an oestrone core containing a different pharmacophore – a sulphamate moiety^32^. The discovery of these compounds based on aryl sulphamate esters became a milestone in the development of derivatives exhibiting extremely high inhibitory activities against STS. Some of them have entered clinical trials with very promising results, especially in the treatment of cancer. Despite the fact that the mechanism of inhibition is still not completely confirmed, it is currently accepted that the inactivation of STS by compounds containing the sulphamate-aryl system occurs *via* a sulphamoyl moiety transfer to the catalytic fGly residue. The current knowledge about a mechanism of STS inhibition by sulphamate-containing compounds has been recently well summarised in the review of *Prof. Barry Potter*^26^.

One of the most active sulphamoylated aryl derivatives, oestrone-3-*O*-sulphamate **5** (*EMATE*) (Figure 3), inhibits the STS enzyme in MCF-7 cells with an IC_50_ value of 65 pM. *EMATE* is a time-, concentration-, and pH-dependent STS inhibitor that acts in an irreversible and active-site-directed manner^33^. Despite the promising STS inhibitory activities of steroid sulphamates (e.g. *EMATE*)^34^^,^^35^, their applications as therapeutic agents in the treatment of hormone-dependent cancers were unfortunately limited because of their very potent oestrogenic properties in rats^36^. Nevertheless, oestradiol 3-*O*-sulphamate **6** (Figure 3) (*E2MATE*), was clinically evaluated (phase II clinical trials) in hormone replacement therapy as an oestradiol prodrug^26^^,^^27^. Furthermore, *E2MATE* is still being considered as a drug candidate for hormone-dependent endometriosis therapy^37^. It was noticed that the treatment of mice with *E2MATE* resulted in a reduction of the development of endometriosis. *E2MATE* inactivated STS in the murine uterus in dose-dependent manner with the lack of an effect on plasma oestradiol levels^38^ and demonstrated clinical advantages in randomised and proof-of-principle phase I clinical trials^37^. Administration of *E2MATE* in monotherapy and in combination with norethindrone acetate (NETA) to healthy women led to a reduction in STS activity in endometrium tissues. Further investigation on its efficacy, safety, pharmacokinetics, and pharmacodynamics are ongoing in phase II clinical trials^26^.

Considering its high STS inhibitory potency, *EMATE* became a lead compound for the development of numerous derivatives with improved biological properties. Woo et al.^39^ synthesised a large library of *EMATE* analogues substituted at the 2- and/or 4-positions (e.g. with halogen atoms, nitro, propenyl, *n*-propyl, and cyano groups) and its derivative with the removed 17-carbonyl motif. An extensive structure–activity relationship (SAR) analysis has been performed – including the influence of electronic effects of substituents on the A-ring and modification of the sulphamate pharmacophore on STS inhibitory activity. It has been noticed, that the presence of electron-withdrawing groups (EWG) in the A-ring affects the potency in a positive way. However, the STS inhibitory effect depended on the type and location of the substituents. For example, higher inhibition was observed for derivatives containing halogen atom at the 2-position. On the other hand, the location of nitro groups at the 4-position was much more favourable. 4-Nitro-*EMATE* **7** (Table 1) was found to be the most potent derivative with IC_50_ values of 0.8 and 0.01 nM in assays with placental microsomes and MCF-7 cells, respectively. Due to a very high STS inhibitory activity, this compound seems to be very promising in the context of further research. Subsequent appropriate *in vivo* investigations should verify the existing potential of 4-nitro-*EMATE* **7** and its usefulness in further clinical trials.

**Table 1.** Examples of steroidal STS inhibitors **7–12**.

General structure	Substituents	Example	STS inhibitory effects	Ref. [year]
	R = H, NO_2_, F, Cl, Br, I, CN, 2-Propenyl or *n*-Propyl; X = CO or CH_2_		IC_50_ values of 0.8 and 0.01 nM (in assays with placental microsomes and MCF-7 cells, respectively)	39 [2012]
	*n* = 0 or 1; R = H, NO_2_, F, Cl, Br, CN, CH_3_, CF_3_, OCH_3_, OCF_3_, *n*-Propyl, *n*-Butyl, *n*-Pentyl, *iso*-Propyl, *tert*-Butyl, Ph, OPh		IC_50_ value of 9 nM (in an assay with purified enzyme)	40 [2012]
	R_1_=H, NO_2_, F or Br; R_2_=Br, CF_3_, *tert*-Butyl, Ph		K_i_ of 1 nM (in an assay with purified enzyme)	41 [2015]
Natural sources	–		IC_50_ value of 10.5 µM (in an assay with JEG-3 cells)	42 [2020]
	–		IC_50_ value of 12.4 µM (in an assay with JEG-3 cells)	42 [2020]
	–		IC_50_ value of 15.7 µM (in an assay with JEG-3 cells)	42 [2020]

Other STS inhibitors based on a steroidal core have been developed recently. A modification of oestrone’s structure by the replacement of a 17-carbonyl group with *N*-17β-arylsulphonamide and *N*-17β-alkylbenzenesulphonamide moieties led to the series of 17β-arylsulphonamides and 17β-alkylbenzenesulphonamides of 17β-aminoestra-1,3,5(10)-trien-3-ol as reversible STS inhibitors^40^. Interestingly, despite the absence of the sulphamate functional group connected to the A-ring in a steroidal core, the obtained compounds exhibited extremely high STS inhibitory properties. The most potent analogue **8** (Table 1) inhibited STS with an IC_50_ value of 9 nM (when evaluated in an assay with purified enzyme). According to the presented SAR analysis, the introduction of alkyl substituents into the arylsulphonamide group resulted in the improvement of STS inhibitory potency – the presence of an *n*-butyl chain was the most favourable (the introduction of an *n*-pentyl substituent resulted in a decrease in the potency). Furthermore, the alteration of an *n*-butyl chain with a *tert*-butyl group occurred to be beneficial for STS inhibitory effect. On the other hand, studies with 3′- and 4′-substituted benzenesulphonamides bearing EWG and electron-donating groups (EDG) showed that 3′-bromo and 3′-trifluoromethyl derivatives were the most potent.

In 2015^41^, the same research group prepared and examined a series of A-ring-substituted 17β-arylsulphonamides of 17β-aminoestra-1,3,5(10)-trien-3-ol as STS inhibitors. The authors noticed that the presence of a nitro group or a fluorine atom at the 4-position of the A-ring resulted in an increase in the potency. In contrast, the derivatives containing these substituents at the 2-position occurred to be less active than A-ring-unsubstituted analogue. The most active derivative **9** (Table 1) demonstrated STS inhibition with K_i_ of 1 nM. Furthermore, the antiproliferative activities (examined using the NCI 60 cell-line panel) of obtained derivatives indicated, that these compounds are promising drug candidates in the treatment of breast and ovarian cancers. Further research dedicated to studies of oestrogenic properties may be useful in the evaluation of their clinical potential.

Recently, Grienke et al.^42^ applied interesting *in silico* method to identify STS inhibitors from natural sources. According to the pharmacophore-based virtual screening of the Traditional Chinese Medicine (TCM) database, triterpenes based on the lanostane structure were taken into account as potential STS ligands. Evaluated compounds were isolated from traditionally used polypores, which are rich in lanostane derivatives. (i.e. *Ganoderma lucidum* Karst., *Gleophyllum odoratum* Imazeki, and *Fomitopsis pinicola* Karst). After sophisticated purification protocols, 3 out of 18 isolated lanostane analogues exhibited STS inhibitory activities (when evaluated in an assay with JEG-3 cells). Piptolinic acid D **10**, Pinicolic acid B **11,** and Ganadrol A **12** (Table 1) inhibited STS with IC_50_ values of 10.5, 12.4, and 15.7 µM, respectively. Despite the moderate STS inhibitory potency, authors indicated that reported compounds are the first STS inhibitors isolated from natural sources. Further application of *in silico* methods might be useful in the identification of more potent natural compounds demonstrating STS inhibitory properties.

**Figure 4.**
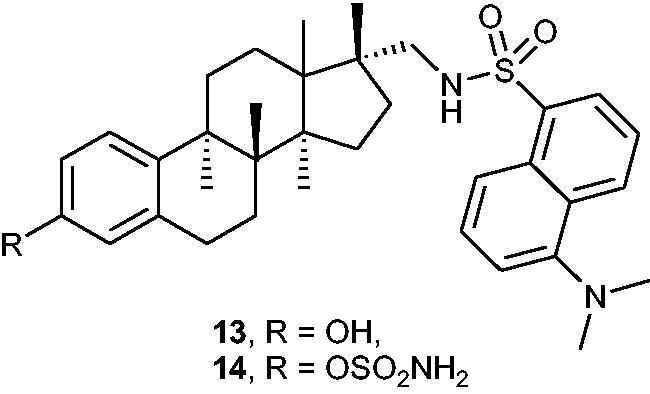
Chemical structures of 17α-dansylaminomethyl-oestradiol **13** and its sulphamoylated derivative **14**.

Maltais et al.^43^ designed fluorescent STS inhibitors based on the steroidal scaffold using quantitative SAR (QSAR) and molecular modelling studies. Based on the previously undertaken research^44^^,^^45^, two newly compounds: 17α-dansylaminomethyl-oestradiol **13** and its sulphamoylated derivative **14** (Figure 4) were designed and synthesised. Both compounds occurred to be effective STS inhibitors, demonstrating IC_50_ values of 69 and 2.1 nM (for compounds **13** and **14**, respectively) in an assay with HEK-293 cells. Interestingly, it was shown that the sulphamoylated derivative **14** may be applicable as an optical imaging tool to investigate intracellular sub-localisation of STS enzyme and inhibitory mechanisms. According to the confocal microscopy analysis, good penetration of the fluorescent inhibitor **14** in cells and its localisation in the endoplasmic reticulum (where STS is localised) have been confirmed.

## 3. Nonsteroidal STS inhibitors

Although the application of steroid derivatives in the development of potential STS inhibitors seemed to be reasonable, the clinical application may be limited in some cases due to their other undesired properties (e.g. the estrogenicity of *EMATE* occurred to be problematic^36^). However, there is insufficient evidence to disparage the use of steroid derivatives in clinical practice. Fortunately, the potential limitations of STS inhibitors based on the steroidal scaffolds can be overcome by the development of nonsteroidal analogues^14^. Theoretically, the change of the steroid core (present in the chemical structure of STS natural substrates) might affect the reduction of the binding abilities of nonsteroidal analogues to the enzyme’s active site. However, a large number of steroid system mimics with promising biological activities has been reported, and until now, the synthesis of nonsteroidal STS inhibitors based on different cores has become a priority in the development of drug candidates with greater clinical applications. To date, derivatives of tyramine, triazole, piperazinyl-ureides, biphenyl, flavones, carboranes, and many others have been reported as potent STS inhibitors (see sections below). Among them, the most promising results have been achieved with the application of coumarin-based analogues^26^. The tricyclic coumarin derivatives effectively mimicked the steroid core of natural STS substrates, and one of them – *Irosustat* – proved to be extremely promising STS inhibitor without having *in vitro* and *in vivo* oestrogenic properties^27^. The discovery of *Irosustat* occurred to be a milestone in the development of potential anticancer therapies based on STS inhibitors^26^.

### 3.1. Nonsteroidal STS inhibitors containing a sulphamate moiety

**Figure 5.**
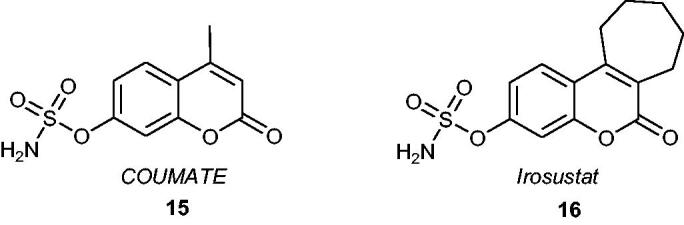
Chemical structures of STS inhibitors based on sulphamoylated coumarin derivatives –*COUMATE* **15** and *Irosustat* **16**.

An important class of compounds that proved to be promising mimics of known steroidal STS inhibitors are sulphamoylated coumarin derivatives. It has been noticed that these analogues exhibited high inhibitory potency against STS and fewer adverse effects, such as much weaker oestrogenic properties than *EMATE*^46^. Similar to *EMATE*, the sulphamoylated coumarin analogues, e.g. 4-methylcoumarin-7-*O*-sulphamate (also known as *COUMATE*) **15** (Figure 5), are classified as irreversible inhibitors that act in a time- and concentration-dependent manner. *COUMATE* exhibited good activity with an IC_50_ value of 380 nM (when evaluated against placental microsomes)^47^. Its modification through the introduction of an additional aliphatic ring gave rise to a wide range of tricyclic coumarin derivatives that mimicked the ABC rings of the natural substrate and demonstrated significantly higher inhibitory effects than *COUMATE*. For example, one of the most promising drug candidates based on a sulphamoylated coumarin core, *Irosustat* **16** (Figure 5), exhibited very potent activity towards STS (IC_50_ value of 8 nM) without having *in vitro* and *in vivo* oestrogenic properties^48^^,^^49^. Woo et al.^50^ published a key paper dedicated to the SAR studies for this first-in-class clinical STS inhibitor. The compound was characterised by favourable bioavailability (however, the bioavailability decreases with increasing doses)^51^, and was orally active and well-tolerated in patients^52^. It has reached clinical trials, and its high therapeutic potential has been proven in several clinical studies^53^^,^^54^. A combination therapy of *Irosustat* with a first-line aromatase inhibitor (phase II clinical studies) in patients with advanced ER-positive breast cancer resulted in clinical benefits with an acceptable safety profile^55^^,^^56^. Furthermore, *Irosustat* has recently shown clinical advantages in early breast cancer^57^ and ER-positive advanced endometrial cancer treatments^58^.

The synthesis of sulphamoylated coumarin derivatives is still one of the main directions in the development of compounds with STS inhibitory activities. Ganeshapillai et al.^59^ synthesised a series of novel STS inhibitors based on bicyclic coumarin sulphamates. Based on the chemical structure of *COUMATE*, a large library of C-3- and C-4-substituted derivatives containing a sulphamate moiety was obtained and a wide SAR analysis was performed. In the case of many synthesised compounds, high STS inhibitory potency was achieved (with the measured IC_50_ values within the nanomolar range). Some of the reported analogues were found to inhibit the STS activity approximately 100–500-fold more effectively than the parent *COUMATE,* with the best examples close in the potency to the clinically evaluated *Irosustat*. Among the synthesised analogues, compounds **17** (a *COUMATE* derivative with an *n*-hexyl chain at C-3 position) and **18** (a *COUMATE* derivative with a benzyl motif at C-3 position) (Table 2) were particularly effective with IC_50_ values of 0.68 and 1 nM in intact MCF-7 cells and 8 and 32 nM in an assay with placental microsomal STS, respectively. Presented results showed that these compounds could represent new leads for potential development after further preclinical studies.

**Table 2.** Examples of nonsteroidal STS inhibitors with sulphamate moiety **17–31**.

General structure	Substituents	Example	STS inhibitory effects	Ref. [year]
	R = H, Cl, CH_3_, (CH_2_)_1-14_CH_3_, *iso*-Propyl, *iso*-Butyl, CH_2_Cl, Ph, (CH_2_)_1-3_Ph, 4-ethylphenyl, 4-methoxyphenyl, CH_2_-adamantyl, *cyclo*-C_6_H_11_ or (CH_2_)_1-2_*cyclo*-C_6_H_11_		IC_50_ values of 0.68 and 8 nM (in an assays with intact MCF-7 cells and placental microsomes, respectively)	59 [2018]
			IC_50_ values of 1 and 32 nM (in an assays with intact MCF-7 cells and placental microsomes, respectively)	
	R_1_, R_2_, R_3_, R_4_, R_5_=H, F, Cl, NO_2_, CH_3_ or OCH_3_; X = (CH_2_)_1-2_, CO		IC_50_ values of 0.13 and 1.35 µM (in an assays with purified enzyme and MCF-7 cells, respectively)	60 [2020]
	R_1_, R_2_, R_3_, R_4_, R_5_=H, F, CF_3_ or OCF_3_		IC_50_ value of 270 nM (in an assay with purified enzyme)	61 [2016]
			IC_50_ value of 270 nM (in an assay with purified enzyme)	
	R_1_, R_2_, R_3_, R_4_, R_5_=H, F, CF_3_ or OCF_3_; n = 0 or 1		IC_50_ value of 180 nM (in an assay with purified enzyme)	62 [2017]
			IC_50_ value of 180 nM (in an assay with purified enzyme); GI_50_ values of 15.9, 8.7, 18.8 and 8.1 µM (in an assay with MCF-7, T47D, SkBr3, and MDA-MB-231 cell lines, respectively)	
	R_1_, R_2_, R_3_, R_4_, R_5_=H, F, CF_3_, OCF_3_ or NO_2_; *n* = 0 or 1; *m* = 8 or 9		IC_50_ value of 2.18 µM (in an assay with purified enzyme)	63 [2017]
	R_1_, R_2_, R_3_, R_4_, R_5_=H, F, CF_3_ or OCF_3_		IC_50_ value of 36 nM (in an assay with purified enzyme)	64 [2019]
			IC_50_ value of 58 nM (in an assay with purified enzyme)	
	R_1_=OSO_2_NH_2_ or OH; R_2_ = H or CH_3_; R_3_ = -, H, CH_3_ or (CH_2_)_1-3_CH_3_; X = C, S or Si		IC_50_ value of 0.17 µM for compound **27** (in an assay with MCF-7 cells)	65 [2014]
	R = CH_3_, (CH_2_)_1-2_CH_3_, Ph, 4-methylphenyl, 4-*tert-*butylphenyl, 4-fluorophenyl, 4-trifluoromethylphenyl, NH_2_, NHCH_3_, N(CH_3_)_2_; *n* = 1 or 2		72.0 and 55.7% inhibition of STS after incubation with 20 and 10 µM of **29**, respectively (in a cell-free assay); 93.9 and 86.1% inhibition of STS after incubation with 20 and 10 µM of **29**, respectively and IC_50_ value of 0.421 µM (in an assay with JEG-3 cells)	66 [2016]
	R = *n*-heptyl, *n*-octyl, *n*-decyl, Ph, 2-methylphenyl, 3-methylphenyl, 2, 3-dimethylphenyl, 3-methoxyphenyl, 4-methoxyphenyl, 4-chlorophenyl, 3, 4-dichlorophenyl, 4-fluorophenyl or CH_2_-3-benzofuranyl; X = H, F or Cl		IC_50_ value of 5.1 nM (in an assay with JEG-3 cell lysate)	67 [2019]
			IC_50_ value of 8.8 nM (in an assay with JEG-3 cell lysate)	

A wide range of novel sulphamoylated derivatives of coumarin has been also synthesised by Hng et al. recently^60^. In the course of undertaken research, the modifications at the 3-position of a coumarin core have been evaluated. SAR analysis indicated, that the attachment of a benzylamino group at the 3-position of sulphamoylated coumarin improved the STS inhibitory activity. Furthermore, the presence of methoxy substituents in the structure of a terminal phenyl ring was significant for STS inhibition. It has been noticed that methoxy *para*-substitution is not associated with the improvement in STS inhibitory potency. On the other hand, the presence of the methoxy substituents at the *ortho*- and *meta*-positions is much more favourable. Among the obtained set of compounds, analogue **19** (Table 2) is found to exhibit the highest STS inhibitory activity against enzyme isolated from human placenta (IC_50_ value of 0.13 µM) and MCF-7 cell lines (IC_50_ value of 1.35 µM). However, this derivative occurs to be still 13 times less potent than *Irosustat* (used as a reference). Despite the moderate STS inhibitory potency of reported compounds, authors indicated that the further modifications of the benzylamino group of compound **19** might be worth exploring.

A strategy involving the introduction of fluorine atoms into the structure of biologically active compounds has been utilised to design new STS inhibitors based on the coumarin core. It is generally known that the introduction of a fluorine atom may affect nearly all physical properties of the drug, as well as its absorption, distribution, metabolism, and excretion. Since 1955, more than 150 fluorinated molecules have succeeded in reaching the market and now are approximately 20–30% of all pharmaceuticals^68^. In 2016, fluorinated 3-phenylcoumarin sulphamates were obtained as STS inhibitors^61^. It was noticed that the presence of a fluorinated phenyl ring at the 3-position of a coumarin core is beneficial for STS inhibitory activity. The most favourable effects were observed for derivatives containing fluorine atoms at the *meta* position in a phenyl substituent. For example, two compounds, **20** and **21** (Table 2), demonstrated the highest activity in an STS enzyme assay with IC_50_ values of 270 nM in both cases. Further investigation on this type of STS inhibitors, involving the synthesis of analogues with an additional aromatic ring attached *via* the amide moiety, led to the development of their more potent *N*-benzoyl and *N*-phenylacetoyl derivatives^62^. The most active compounds, **22** and **23** (Table 2), inhibited a purified STS enzyme with IC_50_ values of 180 nM in both cases. Furthermore, compounds **20–23** also demonstrated cytotoxic properties. For example, analogue **23** demonstrated the most potent antiproliferative activity with GI_50_ values of 15.9 and 8.7 µM for MCF-7 and T47D cell lines, respectively. However, **23** was not selective towards oestrogen-dependent cells and effectively inhibited the growth of ER- and PR-negative cell lines (the GI_50_ values for the SkBr3 and MDA-MB-231 cell lines were 18.8 and 8.1 µM, respectively). In contrast, despite the slightly lower activities of compounds **20** and **21** compared with **23**, analogues **20** and **21** demonstrated selective inhibition of ER- and PR-positive cell line growth. Despite the promising *in vitro* activities of these compounds, their clinical potential should be confirmed in *in vivo* models.

Interestingly, the design of detailed structures of STS inhibitors based on fluorinated 3-phenylcoumarin sulphamates was supported by molecular modelling techniques. Currently, computational approaches have become a key step in the rational development of new potential therapeutics. They are effective in the hit identification and lead optimisation processes as well as structure- or ligand-based virtual screening studies. The molecular docking of small ligands to enzyme/receptor binding sites has been intensively developed since the 1980s (and still remains widely utilised by scientists). Docking calculations may be used as a hit-identification tool, when the structure of a molecular target (or its active/binding site alone) is known as well as during the computational optimisation of the chemical structure of lead compounds before the synthesis of their derivatives^69^. In case of STS inhibitors based on fluorinated 3-phenylcoumarin sulphamates, molecular docking techniques allowed the determination of binding modes of synthesised compounds to the STS structure and the identification of potential interactions between inhibitors and amino acid residues located in the STS active site^61^^,^^62^. For example, the fluorine atoms from some docked derivatives are within short distances to the Arg98 or Thr484 residues, suggesting the presence of additional stabilising interactions that may influence the binding of a potential drug molecule to the enzyme’s active site. Additionally, computational research on geometric optimisation methods for STS inhibitors based on fluorinated 3-phenylcoumarin sulphamates and their influence on the free binding energy with STS has been performed^70^. The obtained results indicated that the MM + and PM7 geometry optimisation methods could be successfully employed for the geometric optimisation of STS inhibitors before their docking procedure and for molecular descriptor calculations.

A fluorination strategy has been recently utilised in the development of novel STS inhibitors based on a tyramine core^63^. The series of obtained *N*-acylated tyramine sulphamates inhibited the STS enzyme at micromolar levels. In the course of undertaken research, the introduction of fluorinated *N*-benzoyl and *N*-phenylacetoyl groups as well as *N*-perfluoroalkanoyl chains into a sulphamoylated tyramine core has been evaluated. Generally, it has been noticed that most of the fluorinated derivatives demonstrated higher STS inhibitory activity than their nonfluorinated counterparts. The IC_50_ value of the most active inhibitor **24** (Table 2) was determined to be 2.18 µM in an enzymatic assay.

Recently, Daśko et al.^64^ developed a new series of potent STS inhibitors based on the fluorinated 4-(1-phenyl-1*H*-1,2,3-triazol-4-yl)phenyl sulphamate core. Almost all of the synthesised compounds inhibited STS at the nanomolar level. During the course of the investigation, the highest inhibitory activities were exhibited by derivatives containing a fluorine atom at the *meta* position of a terminal aromatic ring. The most active analogues, 4-[1-(3,5-difluorophenyl)-1*H*-1,2,3-triazol-4-yl]phenyl sulphamate **25** and 4-[1-(2,3,4-trifluorophenyl)-1*H*-1,2,3-triazol-4-yl]phenyl sulphamate **26** (Table 2), inhibited STS enzyme with IC_50_ values of 36 and 58 nM, respectively, when evaluated in an enzymatic assay (in comparison, the IC_50_ value measured for *Irosustat* in the same assay was 25 nM). According to the molecular docking calculations, the fluorine atoms of compounds **25** and **26** may interact with the Arg98 residue located in the STS active site. This additional interaction may stabilise the inhibitor-enzyme complex resulting in improved inhibitory activity. The presented results indicated that fluorinated 4-(1-phenyl-1*H*-1,2,3-triazol-4-yl)phenyl sulphamates are promising STS inhibitors, however, their clinical potential should be proved in further *in vivo* evaluation.

Kajita et al.^65^ obtained a series of silicon derivatives of diphenylmethane as potent STS inhibitors, which also served as pro-oestrogen antagonists. In the course of the undertaken research, the derivatives with a modified Si-alkyl motif and an aromatic ring system were synthesised. It was noticed that the presence of ethyl groups on a silicon atom was the most favourable (the derivatives containing methyl, *n*-propyl and *n*-butyl were much less active). Among the obtained derivatives, silicon-containing compound **27** (Table 2) showed high STS inhibitory effect (IC_50_ of 0.17 µM when evaluated in an assay with MCF-7 cells). Interestingly, the STS inhibitory activity of compound **27** was significantly higher than the corresponding carbon analogues. Furthermore, the putative metabolite of **27**, compound **28** (Table 2), showed potent ERα-antagonistic activity (IC_50_ value of 29.7 nM) and lacked ERα-agonistic activity.

El-Gamal et al.^66^ reported a new series of arylamides containing sulphonate and sulphamate moieties as novel STS inhibitors. The structures of obtained analogues differed in a pharmacophore region (sulphonate, sulphamate, and *N*-substituted sulphamate groups were evaluated) and in the size of an aliphatic ring (five and six-membered rings were introduced). As it had been predicted, the most potent derivative **29** (Table 2) containing free sulphamate moiety inhibited STS activity by 72.0% and 55.7% at 20 and 10 µM, respectively, in a cell-free assay. Further investigation showed that **29** inhibited (in a dose-dependent manner) 93.9% and 86.1% of STS activity in JEG-3 placental carcinoma cells at 20 and 10 µM, respectively (IC_50_ value of 0.421 µM). Among the derivatives containing aliphatic sulphonate groups, the presence of the ethanesulphonate moiety occurred to be the most favourable. On the other hand, the *para*-tosylate derivative was more active than the other aromatic sulphonates. It was also noticed that the derivatives containing a cyclohexyl ring were significantly more active than the corresponding analogues bearing a cyclopentyl ring in general. The authors indicated that the newly obtained compounds based on a relatively simple structure were promising candidates for future lead optimisation, which could be carried out by an appropriate substitution of the two ring systems, although no article have been published up to date.

Recently, Moi et al.^67^ reported two new groups of piperazinyl-ureido sulphamates as potent STS inhibitors. Initially, the undertaken studies led to the development of a series of 4-(piperazinocarbonyl)-aminosulphamates that demonstrated promising STS inhibitory potency. The development of a few lead compounds resulted in the synthesis of derivatives containing halogen atoms (fluorine or chlorine) within the aryl-sulphamate pharmacophore. The most active derivatives **30** and **31** (Table 2) demonstrated high STS inhibitory activities in an assay with JEG-3 cell lysate (IC_50_ values of 5.1 and 8.8 nM, respectively). In spite of the fact that further inhibitor structure optimisation efforts have been made (such as the introduction of an additional pyrimidinyl ring), enhancement of the inhibitory potency has not been achieved.

**Figure 6.**
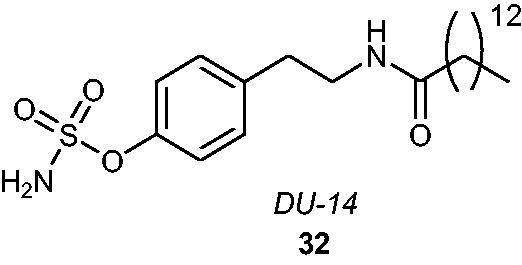
The chemical structure of *DU-14* **32**.

Recently, an interesting *in vivo* study focussed on a derivative of *p*-*O*-sulphamoyl-*N*-alkanoyl tyramine, namely, *DU-14* **32** (containing an *N-*tetradecanoyl chain) (Figure 6), has been described as a potential treatment method of neurodegenerative disorders (e.g. Alzheimer’s disease) in rats^71^. Historically, a series of *p*-*O*-sulphamoyl-*N*-alkanoyl tyramines was synthesised as STS inhibitors by Li et al.^72^. The most active analogue, *DU-14*, promoted an STS inhibitory effect with an IC_50_ value of 55.8 nM when evaluated in an assay with placental microsomes as the enzyme source^72^ and an IC_50_ value of 350 nM when evaluated in an assay with MDA-MB-231 cells^73^. It has been shown^74^ that *DU-14* can enhance the reversal of amnesia by DHEAS, suggesting that STS inhibition could promote the memory-enhancing properties of DHEAS. Yue et al.^71^ reported neuroprotective properties of DU-14 against neurotoxic amyloid β protein (Aβ), suggesting that upregulation of endogenous DHEAS by *DU-14* may be responsible for a beneficial alleviation of impairments in spatial memory and synaptic plasticity induced by Aβ in rats.

### 3.2. Nonsteroidal STS inhibitors containing phosphorus moieties

The initial strategy applied in the designing of STS inhibitors involving the replacement of the sulphate group in natural STS substrates has been recently extended to numerous phosphorus moieties^75^. Because methods for the chemical introduction of phosphorus moieties are widely utilised^76^, medical applications of organophosphorus compounds as drugs or drug candidates in the treatment of various diseases have expanded greatly in recent years. Compounds containing phosphorus groups are used in clinical practice as agents with anticancer and antiviral properties as well as in the treatment of metabolic bone disorders such as osteoporosis^75^^,^^77^. It is known that various phosphorus moieties may react with amino acid functional groups and create electrostatic interactions inside the enzyme active site that may influence the binding of potential drugs to the enzyme’s active site.

Recently, Kozak et al. have synthesised new tricyclic coumarin derivatives containing phosphoric acid residues, dimethyl- and diethylphosphate groups as well as phosphoroamidates and phosphorodiamidates that were able to inhibit STS activity in the micromolar range^78^. The most active derivative **33** (Table 3) inhibited STS with the IC_50_ value of 21.5 µM in an enzymatic assay. Although the mechanism of inhibition is unknown, docking studies suggest the possibility of a phosphate group transfer to fGly75 during the inactivation process. Further development led to the synthesis of thiophosphate analogues with slightly improved inhibitory potencies^79^. The highest activity (in an assay with an isolated enzyme) was exhibited by compound **34** (Table 3), which contained a chlorothiophosphate group (IC_50_ value of 13.3 µM).

**Table 3.** Examples of nonsteroidal STS inhibitors containing phosphorus moieties **33–46**.

General structure	Substituents	Example	STS inhibitory effects	Ref. [year]
	R = Cl, NH_2_, OCH_3_ or OCH_2_CH_3_; X = O or S; *n* = 1, 2, or 3.		IC_50_ value of 21.5 µM (in an assay with purified enzyme)	78 [2014]
			IC_50_ value of 13.3 µM (in an assay with purified enzyme)	79 [2015]
	R = Cl, OH, NH_2_ or OCH_3_; *n* = 1, 2, or 3.		IC_50_ value of 860 nM (in an assay with purified enzyme).	80 [2015]
			IC_50_ value 7.76 µM (in an assay with purified enzyme).	
			IC_50_ value of 6.77 µM (in an assay with purified enzyme)	
	R_1_ = Cl, OH, NH_2_, OCH_3_ or OCH_2_CH_3_; R_2_ = H or CH_2_CH_3_; X = O or S.		IC_50_ value of 46.8 µM (in an assay with purified enzyme)	81 [2015]
	R_1_ = Cl, OH, or OCH_3_; R_2_ = H, F, CH_3_ or CF_3_; X = O or S.		IC_50_ value of 29.0 µM (in an assay with purified enzyme)	82 [2015]
	R = Cl, OH, NH_2_ or OCH_3_.		IC_50_ value of 22.1 µM (in an assay with purified enzyme)	81 [2015]
	R_1_ = H, F, CH_3_ or CF_3_; R_2_ = Cl, OH, or OCH_3_.		IC_50_ value 3.2 µM (in an assay with purified enzyme)	82 [2015]
	R = OH, NH_2_ or OCH_3_; X = O or S; *n* = 10, 11, or 12.		IC_50_ value of 0.39 µM (in an assay with purified enzyme)	83 [2015]
			IC_50_ value of 1.31 µM (in an assay with purified enzyme)	
	R_1_ = CH_3_, CH_2_CH_3_, *iso*-propyl, *n*-butyl, Ph, CH_2_Ph; R_2_ = H or CH_3_; X = O or S.		IC_50_ value of 190 nM (in an assay with purified enzyme)	84 [2016]
			IC_50_ value of 240 nM (in an assay with purified enzyme)	
			IC_50_ value of 200 nM (in an assay with purified enzyme)	85 [2019]

In 2015, the same research group synthesised a series of bicoumarin thiophosphate derivatives as STS inhibitors^80^. Although the mechanism of inhibition remains unclear, molecular modelling studies suggest a completely different manner of binding to the STS active site compared to previously evaluated phosphorus STS inhibitors. The docking experiments indicated that these compounds were able to bind to the STS active site and to fill the whole cavity, preventing the substrate’s access to the catalytic amino acid residues. Both reversible and irreversible STS inhibitors were found within this class of compounds. For example, the hydrogenthiophosphate derivative **35** (Table 3) inhibited STS reversibly, whereas chlorothiophosphate **36** (Table 3) and methylthiophosphate **37** (Table 3) were irreversible inhibitors (measured IC_50_ values of 860 nM, 7.76 µM, and 6.77 µM, respectively, when evaluated in an enzymatic assay).

The same research approach for designing phosphorus STS inhibitors has been utilised by Demkowicz et al. for biphenyl and flavone derivatives. Initially, a series of biphenyl phosphates and thiophosphates was synthesised^81^. Among them, compound **38** (Table 3) inhibited purified STS enzyme with an IC_50_ value of 46.8 µM. When the biphenyl core was replaced by the flavone framework, the inhibitory activities of the obtained compounds were enhanced. For example, compound **39** (Table 3) inhibited STS activity with an IC_50_ value of 29.0 µM in an enzymatic assay^82^. However, when the thiophosphate group was connected to two biphenyl or flavone residues, the inhibitory activities of the obtained compounds were significantly greater; e.g. IC_50_ values of 22.1 and 3.2 µM were measured for compounds **40** and **41** (Table 3), respectively (measured in an assay with STS).

In 2015, a series of *N*-alkanoyl tyramine phosphates and thiophosphates were obtained as potent STS inhibitors^83^. In the course of undertaken research, it was noticed that the highest STS inhibitory effects were exhibited by compounds containing a hydrophobic dodecanoyl carbon chain in the structure of an *N*-alkanoyl tyramine core. In general, the highest STS inhibitory effects were exhibited by analogues containing phosphate moieties. For example, compounds **42** and **43** (Table 3) containing dimethylphosphate or methylphosphoroamidate groups demonstrated the highest inhibitory activities in an enzymatic assay (IC_50_ values of 0.39 and 1.31 µM, respectively). Their mechanism of action is unconfirmed; however, the alkylating properties of methylphosphates suggest the possible methylation of the catalytic fGly75 residue. Interestingly, compound **42** also demonstrated high cytotoxic activity and effectively inhibited the proliferation of MCF-7, MDA-MB-231 and SkBr3 cancer cells (GI_50_ values of 8.80, 6.48 and 5.76 µM, respectively).

Recently, the synthesis of compounds with both sulphamate and phosphorus groups has been proceded by Daśko et al.^84^. A 3-phenyl-coumarin derivative was used as the structural core. Among the obtained series, compounds **44** and **45** (Table 3) exhibited the highest inhibitory activities against purified STS enzyme (IC_50_ values of 190 and 240 nM, respectively). The molecular docking studies indicated that sulphamate moieties in compounds **44** and **45** were located within the STS catalytic region, while phosphoroamidate groups were within a short distance of the Arg98 residue, suggesting the presence of additional stabilising interactions. When the phosphoroamidates were replaced with thiophosphoroamidates, the next group of STS inhibitors was obtained^85^. In this case, the activity of the most active compound **46** (Table 3) (IC_50_ value of 200 nM) was comparable with derivatives **44** and **45** (when evaluated in an enzymatic assay). A more detailed biological evaluation indicated that derivative **44** inhibited STS enzyme by 99.9% in an assay with MCF-7 cells (at 100 nM). Furthermore, toxicology analysis revealed that compounds **44–46** were safe for zebrafish embryos at concentrations exceeding IC_50_ values by up to 12-fold. Further *in vivo* studies might prove their development potential.

## 4. Multitargeting agents with STS inhibitory activities

The development of inhibitors demonstrating multitargeting properties is an alternative approach to STS inhibition. To date, this strategy has allowed scientists to develop novel STS inhibitors able to inhibit other enzymes implicated in the hormone biosynthesis process (e.g. AROM and 17β-HSD). For example, multitargeting agents demonstrating both STS and AROM inhibitory activities are known as dual aromatase-sulphatase inhibitors (DASIs). Initially, the synthesis of the most promising DASIs was based on the already known procedures for AROM inhibitors substituted with a sulphamate pharmacophore. The presence of the sulphamate-aryl system (responsible for STS inhibition) and the 1,2,4-triazole ring in the structure of a single molecule led to compounds exhibiting high STS and AROM inhibitory properties simultaneously. One of the first DASIs, *STX681* **47** (Table 4), a sulphamoylated derivative of the AROM inhibitor YM511, was synthesised by Woo et al.^86^^,^^94^ *STX681* inhibited STS and AROM activities in JEG-3 cells with IC_50_ values of 590 and 0.77 nM, respectively. Furthermore, its high STS and AROM inhibitory activities and promising therapeutic advantages have been also proven in *in vivo* studies^7^^,^^95^.

**Table 4.** Examples of multitargeting agents with STS and/or AROM and/or 17β-HSD1 inhibitory properties **47–60**.

Compound	STS inhibitory effects	AROM inhibitory effects	17β-HSD inhibitory effects	Ref. [year]
	IC_50_ of 590 nM (in an assay with JEG-3 cells)	IC_50_ of 0.77 nM (in an assay with JEG-3 cells)	–	86 [2003]
	IC_50_ value of 5.5 nM (in an assay with JEG-3 cells)	IC_50_ value of 0.5 nM (in an assay with JEG-3 cells)	–	87 [2010]
	IC_50_ value of 830 pM (in an assay with JEG-3 cells)	IC_50_ value of 15 pM (in an assay with JEG-3 cells)	–	88 [2011]
	IC_50_ value of 2.5 nM for compound **50** (in an assay with JEG-3 cells)	IC_50_ value of 0.2 nM for compound **50** IC_50_ value of 0.028 nM for compound **51** (in an assay with JEG-3 cells)	–	89 [2013]
	IC_50_ value of 2.6 µM for the racemic mixture (in an assay with JEG-3 cells)	IC_50_ value of 3.0 nM for the racemic mixture (in an assay with JEG-3 cells)	–	90 [2008]
	IC_50_ value of 3.0 µM (in an assay with JEG-3 cells)	IC_50_ value of 4.2 nM (in an assay with JEG-3 cells)	–	91 [2007]
	IC_50_value of 15.6 nM (in an assay with T47D cells)	–	IC_50_ value of 22.2 nM (in an assay with T47D cells)	92 [2017]
	IC_50_ value of 0.23 µM (in an assay with purified enzyme)	–	IC_50_ value of 0.36 µM (in an assay with purified enzyme)	93 [2018]
	IC_50_ value of 0.89 µM (in an assay with purified enzyme)	–	IC_50_ value of 0.30 µM (in an assay with purified enzyme)	93 [2018]
	IC_50_ value of 2.0 µM (in an assay with purified enzyme)	IC_50_ value of 8.7 µM (in an assay with purified enzyme)	IC_50_ value of 0.095 µM (in an assay with purified enzyme).	93 [2018]
	IC_50_ value of 2.4 µM (in an assay with purified enzyme)	IC_50_ value of 6.0 µM (in an assay with purified enzyme)	IC_50_ value of 0.18 µM (in an assay with purified enzyme)	93 [2018]
	–	–	IC_50_ value of 0.064 µM (in an assay with purified enzyme)	93 [2018]

In 2010^89^, the same research group reported the first highly potent examples of DASIs based on a biphenyl core. The most active derivative, *STX1983* **48** (Table 4), effectively inhibited STS and AROM activities in a cellular assay with JEG-3 cells (IC_50(_*_STS_*_)_ value of 5.5 nM and an IC_50(_*_AROM_*_)_ value of 0.5 nM) and was nonestrogenic. Furthermore, *STX1983* strongly reduced plasma oestradiol levels and potently inhibited liver STS *in vivo*. Further development of DASIs based on the *STX681* and *STX1983* structures has led to a series of hybrid structures with improved dual STS and AROM inhibitory activities (in the picomolar range) in an assay using JEG-3 cells^88^. For example, the extremely active derivative **49** (Table 4) (IC_50(_*_STS_*_)_ value of 830 pM and an IC_50(_*_AROM_*_)_ value of 15 pM) appears to be a very promising candidate for *in vivo* evaluation in hormone-dependent cancer treatments.

Woo et al.^89^ reported extensive research on the synthesis of novel DASIs based on the *STX681* template. The range of chemical modifications in the *STX681* structure was far-reaching and included the relocation and replacement of a halogen atom, introduction of more halogens and replacement of a methylene linker with a difluoromethylene motif. Replacements of a *p*-cyano-phenyl ring with other ring structures and the replacement of the triazolyl group with an imidazolyl group were performed too. Eventually, a series of seventeen new DASIs was obtained. Among them, the fluorinated compound **50** (Table 4) containing an imidazole ring exhibited the highest STS and AROM inhibitory activities (IC_50(_*_STS_*_)_ value of 2.5 nM and an IC_50(_*_AROM_*_)_ value of 0.2 nM when evaluated in an assay with JEG-3 cells). Interestingly, the parent phenol **51** (Table 4) demonstrated an even higher AROM inhibitory activity (IC_50(_*_AROM_*_)_ value of 0.028 nM).

There has recently been extensive progress in the development of novel DASIs, and several other series of compounds exhibiting dual aromatase-sulphatase inhibitory activities have been obtained – specifically sulphamoylated *Letrozole* (e.g. compound **52**, [Table 4])^90^^,^^96–98^ and *Anastrozole* derivatives (e.g. compound **53**, [Table 4])^91^^,^^99^.

Recently, the other enzyme implicated in the hormone biosynthesis process, 17β-HSD, has reached potential therapeutic importance in the treatment of hormone-dependent diseases. Salah et al.^92^ reported the first dual inhibitors of STS and 17β-hydroxysteroid dehydrogenase type 1 (17β-HSD1) as promising therapeutics for oestrogen-dependent diseases. The design of dual STS/17β-HSD1 inhibitors was based on combining structural elements, essential for 17β-HSD1 inhibition, with the sulphamate-aryl pharmacophore (crucial in STS inactivation). Among 12 synthesised compounds, derivative **54** (Table 4) demonstrated the most promising biological properties with well-balanced activities against both the STS and 17β-HSD1 enzymes (IC_50(_*_STS_*_)_ value of 15.6 nM and an IC_50(_*_17β-HSD1_*_)_ value of 22.2 nM when evaluated in an assay with T47D cells). Compound **54** did not exhibit cytotoxic properties or oestrogen receptor interference. Moreover, **54** was also able to effectively reverse E1S/E1-stimulated proliferation of T47D cells (at 400 nM). Authors noticed, that the activity towards STS required an additional substituent within the sulphamate-aryl system and EWG substituents turned out to be the most favourable. On the other hand, strong EWGs impaired STS inhibition by reducing the chemical stability of the sulphamate pharmacophore.

Bacsa *et al*.^93^ synthesised a series of 2- and/or 4-halogenated (chlorinated, brominated, or iodinated) 13β- and 13α-oestrone derivatives as interesting multitargeting agents. Among the obtained analogues, a few derivatives demonstrated dual STS/17β-HSD1 inhibitory properties. For example, two 4-halo-17-oxo-13β compounds **55** and **56** (Table 4) inhibited STS with IC_50(_*_STS_*_)_ values of 0.23 and 0.89 µM, respectively, and 17β-HSD1 with IC_50(_*_17β-HSD1_*_)_ values of 0.36 and 0.30 µM, respectively. Interestingly, two other compounds, namely, 2-bromo- and 2-chloro-13β-estrones **57** and **58** (Table 4), caused inhibitory effects towards three investigated enzymes, becoming triple STS/17β-HSD1/AROM inhibitors (IC_50(_*_STS_*_)_ values of 2.0 and 2.4 µM; IC_50(_*_17β-HSD1_*_)_ values of 0.095 and 0.18 µM; and IC_50(_*_AROM_*_)_ values of 8.7 and 6.0 µM for compounds **57** and **58**, respectively). The authors conducted extensive SAR analysis regarding the influence of type and position of halogen substitution on the inhibitory properties. For example, it was noticed that the inhibitory potential of iodine-containing derivatives of 13β-oestrone depended on the position of an iodine atom. The 2-iodo derivative **59** (Table 4) was a highly specific 17β-HSD1 inhibitor, whereas its 4-iodo analogue **55** demonstrated dual STS/17β-HSD1 inhibitory properties. In contrast, the 2,4-diiodo derivative exhibited weak inhibitory effect against all of the investigated enzymes.

**Figure 7.**
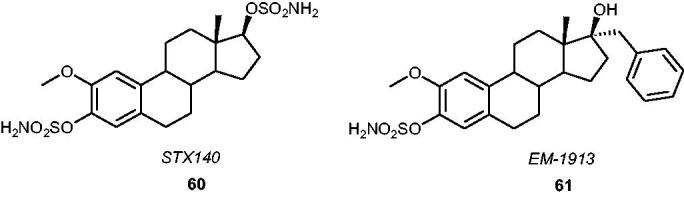
Chemical structures of *STX140* **60** and *EM-1913* **61**.

An extremely interesting drug candidate based on a 2-methoxyestradiol (2ME) structure, *STX140* **60** (Figure 7), has become a very promising multitargeted antitumour agent with a wide range of potential medicinal applications^26^^,^^100^^,^^101^. In addition to its high STS inhibitory potency *in vitro* and *in vivo* (IC_50_ value of 39 nM when evaluated in an assay with placental microsomes)^102^, *STX140* demonstrated numerous interesting properties (e.g. antiproliferative and antiangiogenic activities as well as the ability to induce cell cycle arrest and apoptosis in human tumour xenografts)^103^. It has been observed that *STX140* can inhibit the proliferation of oestrogen receptor-positive (MCF-7) and -negative (MDA-MB-231) cells (with the GI_50_ values of 0.25 and 0.29 µM, respectively)^104^. Therefore, it may be effectively utilised as an STS inhibitor in the treatment of hormone-dependent cancers, however, due to its multitargeting activities, it may be considered in the treatment of tumours with hormone-independent nature as well. Furthermore, favourable pharmacokinetic properties of *STX140*, including good bioavailability and low metabolism in rodents^105^, have proved its major clinical advantages^106^ also for chemotherapy-resistant tumours^107^. In 2013, *in vivo* studies with three breast cancer models^108^ demonstrated that *STX140* had greater anticancer efficacy and therapeutic index as well as reduced neurotoxicity compared to paclitaxel (clinically used for hormone-refractory breast cancer), which may bring significant benefits to patients with breast cancer. Furthermore, a 2-[^11^C]methoxy derivative of *STX140* has been reported as a new potential imaging agent of STS in tumours in the positron emission tomography technique^109^. To date, more sulphamoylated derivatives of 2ME have been developed^110^^,^^111^, including *EM-1913* **61** (Figure 7)^112^, which shows to be a potent STS inhibitor able to block tumour growth (MCF-7 xenograft) in nude mice^113^.

**Figure 8.**
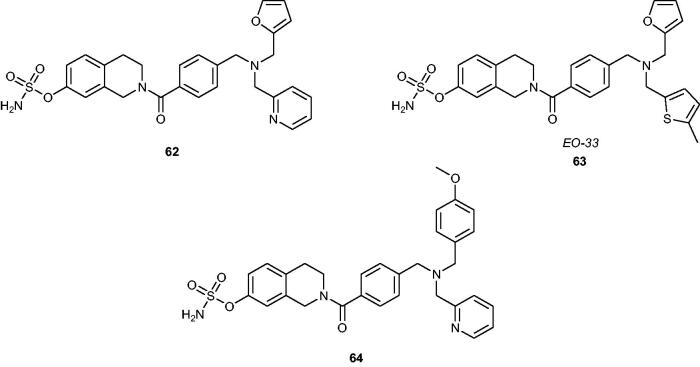
Chemical structures of STS inhibitors with SERM activity **62–64**.

Recently, Ouellet et al.^114^^,^^115^ have reported a new group of STS inhibitors based on *N*-substituted tetrahydroisoquinoline derivatives containing the sulphamate pharmacophore. The most recent research^116^ has demonstrated that such derivatives exhibit both STS inhibitory and selective oestrogen receptor modulating properties. Initially, three parent phenolic compounds, devoid of undesirable oestrogenic activity and toxicity, have been chosen and modified with a sulphamate moiety. These sulphamoylated analogues **62–64** (Figure 8) were potent STS inhibitors (IC_50_ values of 3.9, 8.9 and 16.6 nM, when evaluated in an assay with STS-transfected HEK-293 cells, respectively) without undesirable oestrogenic properties. However, it was noticed that they exhibited moderate antiestrogenic activity. Recent *in vivo* mouse model studies^117^ of one of them (**63**, also known as *EO-33*) have indicated that *EO-33* exhibits a SERM effect and blocks the changes in uterine weight, stimulated by E1S in ovariectomised mice. It has been determined that *EO-33* effectively inhibits STS activity by 81% in the liver and blocks 90% of tumour growth induced by oestradiol sulphate (E2S). Furthermore, no toxic effects (by assessing body weight, liver weight, and liver appearance) have been reported, proving that *EO-33* is a promising nonsteroidal STS inhibitor with SERM activity.

**Figure 9.**
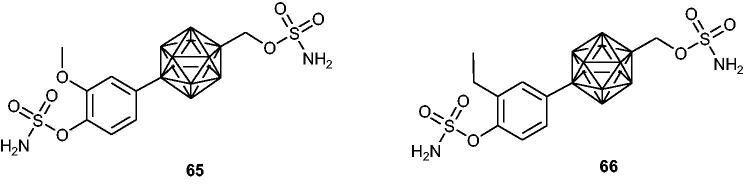
Chemical structures of STS inhibitors based on *p*-carborane-containing sulphamates **65** and **66**.

Kaise et al.^118^^,^^119^ designed and synthesised two series of novel *p*-carborane-containing sulphamates as multitargeting anticancer agents. Obtained compounds possessed both sulphamoylated phenol and alcohol systems at the same time. In the course of undertaken research, it was noticed that the former are much more effective in STS inhibition than the latter. Among the obtained compounds, a few analogues demonstrated high STS inhibitory properties with IC_50_ values in the nanomolar range (when evaluated in an assay with MCF-7 cells). Two of the most promising ones, **65** and **66** (Figure 9), displayed high STS inhibitory effects with IC_50_ values of 0.3 and 1.8 nM, respectively. Interestingly, these compounds demonstrated high antiproliferative activities against significant number of human cancer cell lines, making them promising therapeutic agents for anticancer treatment (a screening panel of 39 human cancer cell lines [e.g. breast, central nervous system, colon, lung, melanoma, ovary, kidney, and stomach] was utilised). Furthermore, both compounds **65** and **66** exhibited tubulin-polymerisation-inhibitory (TPI) activities.

## 5. Conclusion

Despite the development of many treatment strategies, cancer remains one of the most important medical problems that modern medicine has to face. Considering that numerous carcinomas exhibit a hormone-dependent nature, it is rational to design agents that can block hormone biosynthesis processes. The administration of small molecules, exhibiting inhibitory activities against enzymes implicated in hormone biosynthesis may reduce the concentrations of these in tumour tissues and consequently may limit the oestrogenic stimulation of cancer cell growth. Currently, STS is recognised as an extremely promising molecular target in the development of effective agents with high therapeutic potential in the treatment of hormone-dependent cancers. Scientists announce discoveries of new molecules demonstrating STS inhibitory properties every year. Most of them are based on nonsteroidal cores and contain a sulphamate moiety. However, current studies have demonstrated that derivatives with other functional groups (e.g. phosphates and thiophosphates) also exhibit high STS inhibitory activities. Despite the fact that organic synthesis is the most important source of novel STS inhibitors, it is worth noting that a few of recently reported compounds are of natural origin. The research on the natural-based compounds and their modified synthetic analogues may lead to identification of new ones with interesting physicochemical and biological properties. Furthermore, the design process as well as the identification of the potential STS inhibitors are currently more often supported by the computational approaches, e.g. virtual screening, QSAR analysis, and molecular docking studies.

To date, some of the reported compounds have been evaluated in clinical trials (e.g. *Irosustat* and *E2MATE*) and they seem to be very promising as drug candidates. However, none of them has reached pharmacies. Therefore, the identification and synthesis of novel compounds demonstrating STS inhibitory activity as well as the evaluation of the biological influence and safety of known STS inhibitors are crucial for the development of effective treatment methods of hormone-dependent cancers. The discoveries in the synthesis of compounds, which mechanism of action is based on some innovative multi-targeting approaches, have reached the key importance. The recent research has indicated that it is possible to design multitargeting agents that can effectively inhibit STS and other enzymes involved in the hormone biosynthesis pathway (e.g. AROM and 17β-HSD1) or SERM activity. This direction in the development of novel drug candidates with multitargeted biological properties may enhance the efficacy of novel treatment methods. On the other hand, a combination therapy of STS inhibitors with agents targeting other enzymes involved in the hormone biosynthesis process may affect potential clinic advantages (as it has been noticed in the combination therapy of *Irosustat* and AROM inhibitor). Potentially, further clinical benefits may be received in the administration of the STS inhibitors with other anticancer agents, which mechanism of action is not associated with the steroidogenesis process. In this matter, the wide possibilities for improving the efficacy of potential therapies seem to be still achievable. For example, novel compounds chemically constructed of STS inhibitors and other anticancer agents (additionally linked to natural or synthetic carriers providing the selective uptake by the cancer cells) may increase efficacy, selectivity of the therapies and may reduce potential side effects connected with the administration of some agents. On the other hand, the most recent studies have indicated that newly developed compounds (e.g. derivatives exhibiting fluorescent properties) may be applied not only as STS inhibitors but also as optical imaging tools to investigate intracellular sub-localisation of STS enzyme and inhibitory mechanisms.
